# Pregnancy Complications After *Dobbs*: The Role of EMTALA

**DOI:** 10.5811/westjem.61457

**Published:** 2024-01-04

**Authors:** Kimberly Chernoby, Brian Acunto

**Affiliations:** * George Washington University School of Medicine and Health Sciences, Department of Emergency Medicine, Washington, D.C.; † Atlanticare Regional Medical Center, Department of Emergency Medicine, Galloway, New Jersey

## Abstract

In June 2023, the Supreme Court declared that there was no longer a right to abortion under the federal constitution. This decision has allowed states to promulgate different restrictions on abortion, many of which implicate the practice of emergency medicine. An abortion is defined as a “medical intervention provided to individuals who need to end the medical condition of pregnancy” and includes care such as termination of an ectopic pregnancy and induction of labor for previable preterm premature rupture of membranes—interventions that emergency physicians either perform or rely on the assistance of consultants to perform. State bans on abortion must be evaluated against duties under the Emergency Medical Treatment and Labor Act, a federal law that preempts state law. In this paper we examine the conflict between state and federal law as it applies to emergency abortion care and describe how emergency physicians can continue caring for patients.

## DISCLAIMER

This paper does not constitute legal advice nor should it be construed as such. Instead, this paper is for educational purposes only and identifies for physicians key legal points to consider when caring for patients with pregnancy complications in a state with abortion restrictions as a therapeutic option. Only an attorney licensed in the state you are practicing can give you legal advice on this matter.

## PREGNANCY COMPLICATIONS AFTER *Dobbs*: THE ROLE OF EMTALA

Emergency physicians are trained to take care of patients with any complaint at any time of day. This core responsibility includes taking care of pregnant patients. However, what used to be routine medical care has devolved into a rapidly shifting paradigm after a US Supreme Court decision in June 2022 ended the federal constitutional protection of the right to abortion. Since *Dobbs v Jackson Women’s Health Organization*, abortion restrictions have proliferated across states. These state laws are being interpreted in some instances as restricting the medical care that can be provided to pregnant patients—signaling a steep departure from the standard of care. However, despite changing state abortion laws, the Emergency Medical Treatment and Labor Act (EMTALA) still requires that emergency physicians provide stabilizing treatment for patients with emergency medical conditions. This federal law preempts conflicting state laws; so even in the face of state abortion restrictions, physicians need to be cognizant of their duties under EMTALA to render stabilizing medical care, which in some circumstances includes emergency abortion care.

## PREGNANCY COMPLICATIONS IN THE EMERGENCY DEPARTMENT

Pregnancy complications are the fifth most common reason women between ages 15–64 visit emergency departments (ED) in the United States.^1^ As many as 84% of pregnant people visit an ED during pregnancy.^2^ While some emergency physicians have the benefit of an in-house obstetrician (OB), many do not. In the last 13 years, 217 rural hospitals have closed their labor and delivery units.^3^ This means that an increasing number of emergency physicians are responsible for managing pregnancy complications, including discharging and transferring patients appropriately, without the support of an in-house OB.

## THE LEGAL HISTORY OF THE RIGHT TO ABORTION IN THE UNITED STATES

In 1973, the US Supreme Court recognized a federal constitutional right to abortion in *Roe v Wade*.^4^ In an earlier case related to the right to birth control, the Supreme Court found that people have a right to privacy in their intimate relationships.^5^ In *Roe* the Court found that this right to privacy also included the right to abortion. This right was affirmed in several subsequent decisions and was said to extend to the point of fetal viability. Under the legal framework that emerged over the course of 50 years, states could pass restrictions on abortion such as waiting periods and mandatory ultrasounds unless the restriction posed an undue burden on abortion access, but patients ultimately still had a right to obtain an abortion up until the point of fetal viability. This meant that when pregnant patients presented with emergency medical conditions prior to fetal viability, physicians could offer abortions as part of emergency medical care.

In 2018, Mississippi enacted a 15-week abortion ban.^6^ On its face, this law violated prior Supreme Court holdings, and usually such a law would be struck down as unconstitutional. However, the challenge to this law gave the Supreme Court the opportunity to revisit its decision in *Roe*. In *Dobbs v Jackson Women’s Health Organization*, an abortion clinic in Mississippi challenged the state’s 15-week abortion ban as unconstitutional. When this case made its way to the Supreme Court, the Court had to decide whether its prior decision related to abortion (*Roe*) should stand.

Stare decisis is the principal that courts will adhere to prior decisions, also known as precedents.^7^ In this instance, it would mean that the Supreme Court would strike down the Mississippi law because it had already decided that the US Constitution protected the right to abortion up until the point of viability, which is well beyond 15 weeks of gestation. The Supreme Court has also held, however, that in very extraordinary circumstances it will not apply stare decisis, and instead it will overrule precedent. That is what happened in *Dobbs* when the Court overturned *Roe* and held that the right to privacy in the US Constitution does not protect the right to abortion.

The Tenth Amendment to the US Constitution says that if a power is not delegated to the federal government, then it is generally reserved to the states. Since there is no longer a right to abortion under the federal constitution, states have been able to make their own laws pertaining to abortion. Some states already had state constitutional protections for the right to abortion at the time of *Dobbs*, and others have since acted to protect the right to abortion, with three states enshrining the right to abortion in state constitutions. More commonly, though, *Dobbs* has led to state restrictions on abortion, including abortion bans. *Dobbs* allowed previously existing but not enforced bans to go into effect, while in other instances state legislatures have passed new abortion bans. There are currently 15 states with near-total bans (three of which are not in effect pending litigation) and four states with gestational bans that previously would have been unconstitutional.^8^

Some abortion bans that have been enacted since *Dobbs* are less restrictive than others. For example, some bans apply to all gestational ages while others only apply to later gestational ages. Some bans include an exception or affirmative defense in cases where the health and life of a pregnant person is in jeopardy, while others only include an exception or affirmative defense for the life of a pregnant person. Other exceptions may include rape, incest, or fetal anomalies. Shown in the Table are abbreviated examples of two state abortion bans and associated exceptions. State A illustrates a less restrictive ban and State B illustrates a more restrictive ban. These are excerpts and do not include the full scope of the abortion bans, such as language related to aiding and abetting in the provision of an abortion.

**Table.** Examples of abortion bans.

Examples of abortion bans
State A
(a) It shall be unlawful for any person to intentionally perform or attempt to perform an abortion except as provided for by subsection (b).
(b) An abortion shall be permitted if an attending physician licensed in State A determines that an abortion is necessary in order to prevent a serious health risk to the unborn child’s mother.
State B
(a) Every person who performs or attempts to perform an abortion as defined in this chapter commits the crime of criminal abortion.
(b) It shall be an affirmative defense to prosecution under … this section…that: (i) The physician determined, in his good faith medical judgment and based on the facts known to the physician at the time, that the abortion was necessary to prevent the death of the pregnant woman.

## RELEVANCE OF ABORTION TO EMERGENCY MEDICINE

The term “abortion” has a clinical meaning that is very broad. However, the term has been stigmatized and so is often underused. Frequently, physicians provide care that is technically an abortion, but they do not characterize the care as abortion care.^9^ It is unclear whether this failure to accurately characterize care is deliberate, or because physicians do not know that the care they are providing is an abortion. This is problematic because physicians may not understand that they are potentially providing care that is banned under relevant state law. Additionally, patients also understand the term abortion to mean very different things, adding complexity to the dialogue between physicians and patients when managing pregnancy complications.^10^

Abortion is defined by the American College of Obstetricians and Gynecologists as “a medical intervention provided to individuals who need to end the medical condition of pregnancy.”^11^ State laws can be similarly broad. For example, one state defines abortion as “the termination of human pregnancy with an intention other than to produce a live birth or remove a dead fetus.”^12^ Many states specifically carve out procedures such as termination of an ectopic pregnancy with language that includes in its definition, for example, “an act is not an abortion if the act is done with the intent to…remove an ectopic pregnancy.”^13^ It is important that emergency physicians be aware of the broad technical definition of abortion, so that they can be cognizant of applicable laws.

Although not intuitive, these definitions includes interventions such as providing methotrexate for an ectopic pregnancy or induction of labor for previable preterm premature rupture of membranes (PPROM).As methotrexate terminates an ongoing pregnancy, this constitutes an abortion.^14^ Similarly, management of previable PPROM can include either a dilation and evacuation (D&E) done in an operating room or an induction of labor (induction abortion), both of which are forms of abortion. In 2012, the American College of Emergency Physicians issued a clinical policy related to early pregnancy complications that included administration of methotrexate in the ED for ectopic pregnancies.^15^ This means that emergency physicians may find themselves providing care that constitutes an abortion, or consulting colleagues for care that constitutes an abortion.

This is particulary important for emergency physicians to understand because there are instances in which a patient may present to an ED with an ectopic or PPROM and not yet be in extremis. In such a situation, an abortion ban with no exception for the health of a mother may be interpreted as banning an abortion for these patients, even though an abortion is considered the standard of care. Patients are being denied appropriate treatment and suffering as a result. A Texas hospital has stopped offering emergency abortion care for patients with previable pregnancy complications in response to the state’s abortion ban, and the morbidity rate for patients has gone from 33% to 57%.^13^ This is not limited to one state but is in fact happening across the country:

• In August 2022, a patient presented to hospitals in Missouri and Kansas with PPROM at 17 weeks gestation. She was denied emergency abortion care at both hospitals and was sent home to watch for signs of sepsis, hemorrhage, or active labor. She traveled across state lines to obtain the abortion she needed.^16^ The Department of Health and Human Services (HHS) investigated two hospitals that did not provide abortion care (D&E or induction of labor) that she needed, and both hospitals were found to have violated EMTALA for not providing the stabilizing care (abortion) that the patient needed.^17^ ^,^ ^18^ ^,^ ^19^
• In December 2022, two women went to two separate Florida hospitals, both with previability PPROM. One patient was discharged without the emergency abortion she needed and delivered the fetus out of hospital the following day. She required emergency surgery and was subsequently admitted to the intensive care unit in critical condition. The other patient was repeatedly discharged with return precautions, including when she was four centimeters (cm) dilated, and was ultimately admitted for spontaneous delivery when she was in active labor. Both patients required subsequent surgeries that may limit their future fertility.^20^
• In February 2023, a patient presented to a hospital in Oklahoma and was diagnosed with a malignant molar pregnancy that required an abortion. She was not offered the abortion she needed but was told to wait in the parking lot until she decompensated so that she could receive life-saving care in a timely manner. She traveled across state lines to obtain the abortion she needed.^21^
• In March 2023, five women sued the state of Texas for their abortion ban in light of harms suffered from being denied critical abortion care. Two of the women suffered previable PPROM and were denied D&Es or inductions of labor.^22^

These cases are not happening in isolation. There are over 50 reports from across the country of patients receiving different iterations of sub-standard care because of state abortion bans, including being inappropriately discharged with PPROM only to return septic, or being discharged with ectopic pregnancies implanted in C-section scars only to later require a hysterectomy.^23^ Emergency physicians may be liable for this care when they are the physician discharging the patient, especially given requirements under EMTALA.

## EMERGENCY MEDICAL TREATMENT AND LABOR ACT

EMTALA is a federal law enacted in 1986 to ensure that patients have access to emergency medical care regardless of their ability to pay.^24^ EMTALA requires that any patient who presents to an ED is offered a medical screening exam.^25^ The medical screening exam must be performed to determine whether an emergency medical condition exists.^26^ An emergency medical condition is defined under EMTALA as “a condition manifesting itself by acute symptoms of sufficient severity (including severe pain) such that the absence of immediate medical attention could reasonably be expected to result in placing the individual’s health (or the health of an unborn child) in serious jeopardy, serious impairment to bodily functions, or serious dysfunction of bodily organs.”^27^ If an emergency medical condition is present, then a physician must provide either stabilizing care within the capacity of the hospital or risk-minimizing medical treatment and an appropriate transfer to another medical facility if such stabilizing care is not available.^28^ It is important to note that the Supreme Court has previously held that the motive behind a transfer does not matter when determining whether a transfer of care is within the bounds of EMTALA.^29^

If a physician, including an emergency physician or on-call consultant, fails to provide required stabilizing care under EMTALA, they can be personally liable for fines up to $119,942 for each violation.^30^ ^,^ ^31^ This is in addition to fines levied on the hospital, as well as a personal civil action patients may initiate against hospitals. If an on-call physician is not available or refuses to provide the needed care, the emergency physician can discharge their EMTALA duty by arranging an appropriate transfer, although the on-call physician will still be liable for violating EMTALA. EMTALA is relevant to the care of pregnant patients experiencing complications because it requires stabilizing treatment and care, which in some cases is an abortion.

## EMTALA AND STATE ABORTION BANS

Article VI, Clause 2 of the US Constitution establishes what is known as the Supremacy Clause.^32^ The Supremacy Clause of the Constitution means that where there are conflicting federal and state laws, federal law controls. In other words, federal laws generally preempt conflicting state laws. In the instance of abortion bans, that means that the requirement to provide stabilizing treatment under EMTALA, including emergency abortion care, preempts conflicting state laws such as abortion bans. In July 2022, the HSS issued guidance to clarify that EMTALA requires abortion care despite contrary state laws.^33^ Specifically, the guidance states:

“If a physician believes that a pregnant patient presenting at an emergency department is experiencing an emergency medical condition as defined by EMTALA, and that abortion is the stabilizing treatment necessary to resolve that condition, the physician must provide that treatment. When a state law prohibits abortion and does not include an exception for the life of the pregnant person — or draws the exception more narrowly than EMTALA’s emergency medical condition definition — that state law is preempted.”

Shortly after this guidance was issued, two federal courts considered the relationship between EMTALA and state abortion bans.^34^ A federal court in Idaho found that ETMALA preempted the state’s abortion ban, and enjoined the state’s abortion ban insofar as it conflicted with EMTALA, noting the role of the Supremacy Clause.^35^ Specifically, the state had a narrow exception that only allowed abortions to be done to prevent death but did not provide an exception to protect the health of the pregnant person. The court found that this narrow exception conflicted with the requirements of EMTALA to provide emergency abortion care when the health or organ function of a patient was threatened and as such was preempted. The court issued an injunction against the state abortion ban.^36^ However, the law was subsequently amended to exclude treatment of ectopic and molar pregnancies from the ban, and the state Supreme Court wrote in an opinion upholding the abortion ban that the law did not apply to nonviable pregnancies.^37^ ^,^ ^38^ Subsequently, a panel of judges from the Ninth Circuit Court of Appeals reversed the original circuit court decision holding that the Idaho abortion ban as amended, and with the state Supreme Court’s clarifying decision, did not conflict with EMTALA.^39^

Most recently, the Ninth Circuit Court of Appeals issued an order vacating the panel decision, and the case will be reheard in front of the entire Court (en banc).^40^ In a challenge brought by the state of Texas, a federal court in Texas ruled that the Texas abortion ban was not in conflict with EMTALA and, therefore, the state law was not preempted. Consequently, the HHS guidance regarding emergency abortion care was enjoined in Texas and against members of certain medical societies that joined that lawsuit. Both the Idaho and Texas cases are on appeal.^41^ As states choose to define medical emergencies differently, and have differing thresholds that trigger exceptions to abortion bans, this tension between state law and EMTALA will continue to be an issue for practicing physicians.

## MANAGING PREGNANCY COMPLICATIONS AFTER *Dobbs*

Given the ongoing tension between state abortion bans and EMTALA, it is essential for physicians to be aware of any relevant laws in the geographic area they are practicing. When providing emergency medical care for a pregnant patient who needs an abortion, understanding that the term abortion may include care physicians don’t routinely think of as an abortion, the first thing to consider is whether the state has an abortion ban and whether it applies to the gestational age of the pregnancy in question. If a physician is practicing in a state that does not have an abortion ban, then they can provide whatever care is indicated. If a physician is practicing in a state that has an abortion ban and it applies to the patient’s pregnancy based on gestational age, then the next question to consider is whether any exceptions or affirmative defenses apply. For example, if a patient who is seven weeks pregnant presents with a tubal ectopic pregnancy, and the state has a law banning abortion after 12 weeks, then the abortion ban does not apply to the patient and the physician can proceed normally. If there was a six-week abortion ban, then the physician would need to consider whether any of the exceptions apply.

To know if any exceptions to a state abortion ban apply to a patient, a physician will need to be familiar with the abortion laws in their state. Broadly, the two exceptions that are relevant to emergency physicians are exceptions for health and/or life of the pregnant person. Other exceptions that may be relevant would include exceptions for specific diagnoses such as ectopic pregnancy or PPROM. An example of applying an exception for the life of a pregnant person would be if a patient has a ruptured ectopic pregnancy and is unstable. Even if the pregnancy still has cardiac activity, that patient would meet an exception that allows for abortion to protect the life of the pregnant person. The above examples from State A and State B both illustrate such exceptions (A) or affirmative defenses (B).

The legality of management vis-à-vis state abortion bans becomes more unclear when the patient is stable. For example, if a patient has an early ectopic pregnancy that is not ruptured, some may argue that giving methotrexate (which will cause an abortion) would be permissible if there is an exception for both the health and life of a pregnant person, such as State A, but may not be permissible in a state with a narrow exception only to protect the life of the pregnant person, such as State B. In a state that only has a carve-out for the life of the pregnant person, like State B, the recommendation may be to discharge the patient until they become unstable and meet the criteria for an exception to protect the life of the pregnant person. However, state laws should be read broadly to protect abortion care when necessary to protect a pregnant person, and in any event EMTALA requires emergency abortion care in any state—even where the emergency medical provision in a state abortion ban appears narrower than EMTALA’s definition of emergency medical condition.

This is where understanding EMTALA becomes critical. Even if a physician is practicing in a state with a narrow exception that only permits abortion when needed to save the life of a pregnant person, the physician is still obligated to comply with EMTALA. Under EMTALA, treatment is required if there is a threat to a patient’s health, bodily functions, or the function of a bodily organ without treatment. Given that EMTALA preempts conflicting state abortion bans, that means that an abortion must be offered to patients, if indicated, when there is a threat to a patient’s health, bodily function, or the function of a bodily organ even if such an abortion would be impermissible under state law. Failure to provide emergency abortion care in these situations would constitute an EMTALA violation.

## PREPARATION IS KEY

Standard of care does not change across state lines, but we know that the care being offered to patients differs depending on local law because of the proliferation of abortion bans since *Dobbs*. Patients with pregnancy complications can present critically ill or with a condition that could rapidly deteriorate, such that immediate care us required. That is not the best occasion to be navigating a state abortion ban for the first time. Instead, preparation is critical. Emergency physicians working in states with abortion bans should meet with stakeholders including OB/GYN, whether in house or at the local hospital where pregnant patients are referred, hospital counsel, risk management and others to establish clinical policies that address the management of patients with emergent pregnancy complications. These clinical policies should be mindful of hospital and clinician obligations under EMTALA as well as state law.

Emergency physicians are compelled by EMTALA to provide stabilizing treatment, to engage consultants as needed to provide stabilizing treatment, and to transfer patients when needed. In cases of pregnancy complications, this often means consulting with in-house OB or transferring a patient to a hospital that has OB services. In the case of methotrexate therapy for ectopic pregnancies it can also mean directly providing the care. Emergency physicians must remember that if a consultant refuses to evaluate and treat a patient, or refuses transfer, based on a state abortion ban, then all parties involved may be violating EMTALA. Instead, patients should receive standard-of-care treatment including abortion care when indicated. Just as we would not accept recommendations from a surgeon to discharge a patient with an 8-cm, symptomatic aortic aneurysm and to instruct the patient to return for definitive treatment when the aorta is ruptured, emergency physicians should not accept recommendations to discharge patients with emergent pregnancy complications without treatment.
